# The application of 3D printing in anatomy education

**DOI:** 10.3402/meo.v20.29847

**Published:** 2015-10-16

**Authors:** Yousef AbouHashem, Manisha Dayal, Stephane Savanah, Goran Štrkalj

**Affiliations:** 1 Faculty of Science and Engineering, Macquarie University, Sydney, Australia; 2 School of Science and Health, Western Sydney University, Campbelltown, Australia; 3 Faculty of Human Sciences, Macquarie University, Sydney, Australia; 4 Faculty of Science and Engineering, Macquarie University, Sydney, Australia. Email: goran.strkalj@mq.edu.au

Modern medical education relies on a wealth of resources as one of the key elements in developing students’ clinical competencies. Acquiring these resources represents a considerable challenge for many medical schools, not only for financial but also a variety of other reasons, including ethical, legal, and cultural. Obtaining human tissue, in particular, faces many constraints, which, in some countries and cultural circles, create serious problems for medical educators. However, modern technology seems to offer solutions in acquisition of at least some of the resources. Among the new technologies that have in recent years entered the biomedical fields in research, practice, and education are the rapid prototyping techniques, particularly as applied in three-dimensional (3D) printing (1, 2).

In this paper, using an example from Australia's Macquarie University and Western Sydney University, we outline how 3D printing can be successfully used in anatomy education. Utilisation of 3D printing is a part of the long-term educational project at both these institutions, including fields as diverse as medicine, arts, and sciences. In anatomy, the first stage of the project, described here, focused on producing highly accurate 3D prints of human bones.

## Educational resources

Acquiring in-depth knowledge of human structures and the ability to apply this knowledge within the clinical context is an imperative in many medical disciplines (3, 4). At Macquarie University and Western Sydney University anatomy instruction is provided to a diverse group of students enrolled in medical and science degrees. Teaching is carried out through the utilisation of a variety of resources. In the anatomy laboratory various medical images are utilised, together with anatomy models, prosected cadavers, and human bones. To the existing resources, a new addition was made this year – 3D prints of selected bones. These prints were made from the 3D surface scans of the bones from the Macquarie University Skeletal Collection.

There were several reasons for the decision to start the 3D-printing project with human bones. First, bones almost naturally lend themselves for printing as they are generally monochromatic and made of hard tissue. Technically, this makes them the easiest component of the human body to duplicate in 3D printing, with high levels of accuracy, preserving both visual and haptic values of the real tissue.

Second, obtaining bones for anatomical study is a complex process. Although obtaining cadavers for anatomy education (with time restrictions because of the legal requirement to cremate the body within the maximum of 8 years) does not represent a problem, thanks to the well-developed whole body donation programmes in Australia, the acquisition of bones for a permanent collection is constrained by ethico-legal norms. The main resources used for teaching osteology are thus anatomical bone models and bones already acquired for the university's collection (mainly past donations from health professionals’ private collections). Although both institutions have large collections of models, these are not sufficient for teaching purposes. Indeed, anatomical models, even those of high quality are rather schematic and do not show the range of variation present in different human populations in health and disease (5, 6). The Macquarie collection of human bones, however, although relatively small, contains a considerable number of anatomical variants and pathologies that are essential in education. Many of these valuable bony elements are rare; some are quite fragile and were up to now used only in demonstrations, with very limited opportunities for students to handle and examine them directly. Through the advent of 3D printing, students are now able to handle and examine their exact replicas which are printed in several copies.

Finally, an important reason for printing bones is financial in nature. Once the appropriate infrastructure is in place, printing is the most cost-effective way of acquiring a large and representative osteological sample.

## 3D printing and its application

3D printing has, in the last two decades, been successfully utilised in different medical fields, including education. In anatomy, high-quality 3D-printed replicas of cadaveric material were recently produced for teaching purposes (7, 8). Following these pioneering enterprises, in the project described here, 3D prints of bones were produced and introduced to students in anatomy education. This was accomplished in several steps through a community of practice between the two participating universities (9). The project was a continuation of an existing collaboration between the two universities in the field of biomedical education. It capitalised on the existing resources and infrastructure (3D surface scanners, printers, skeletal collections, etc.), as well as the expertise at both universities, enabling production of high-quality scans and prints and increasing the number and variation of the osteological samples.

Bones were scanned using the handheld Artec Spider 3D surface scanner. The obtained 3D images in themselves represent useful educational tools as they can be easily downloaded from the universities’ databases and manipulated by students (e.g., enlarged, viewed from different perspectives, and annotated). 3D replicas were then printed at both universities using the following 3D printers: Objet Connex, Mojo, and the MakerBot Replicator. Osteometric analysis was carried out and revealed that there were no significant differences in the shape and dimensions of the prints when compared to the real bones upon which they were made. Similar results were obtained in an earlier study that examined the accuracy of the 3D prints of cadaveric material (8).

The obtained 3D prints of bones were then used in anatomy laboratories at both participating universities. Students had a chance to handle and examine all specimens, including the rare and fragile elements previously not available for inspection. It is planned that 3D prints will also be utilised in pathology and radiology classes at both Macquarie University and Western Sydney University. Currently, another study is underway that focuses on the usefulness of these 3D prints in education. The study includes an investigation of students’ and teachers’ perceptions of the educational value of the 3D prints and projects in which volunteers will complete a series of anatomy tests on the anatomy models, 3D prints, and real bones.

Future plans regarding this stream of 3D printing include scanning and printing of other anatomical structures, particularly those not easily visible on cadavers and difficult to visualise. These will include, but not be limited to, small elements (bones of the middle ear), cavities (sinuses, ventricles of the brain), and anatomical variations and pathologies. These 3D prints will be used in anatomy and a number of clinical subjects.

Furthermore, as 3D-printing technologies evolve and continue to reduce in cost, alternative printers and techniques will be explored. A range of options, such as vat photopolymerisation, binder jetting, and powder bed fusion, will allow printing of structures that more closely mimic the original resource. For example, choice of materials and printer options can customise anatomy prints to match the weight of the original model. In addition, some newer 3D printers such as the CubePro C and the Connex series enable affordable 3D prints in full colour.

Another improvement that can be made in this preliminary study is to reduce the effort of using a handheld 3D surface scanner and instead use a micro-CT like the Quantum GX creating images that can be converted to .STL files ready for 3D printing. This technology lends itself well to human bones as images are of very high resolution and the scans produced include the internal structure of the bones which will also then mimic the actual weight of human bones if an appropriate consumable is used for printing. This also eliminates the extra step of scanning bones and then using the software to create a 3D file, thus saving time and increasing the resolution for printing.

## Conclusion

3D-printed bones are being successfully applied in anatomy education at Macquarie University and Western Sydney University. The application of 3D prints will further ramify and expend into other subjects. Furthermore, the 3D-printing project will soon involve other anatomical structures, particularly those that are difficult to observe and manipulate. The resources for medical education will continue to develop and evolve following both technological development and increasing educational demands for the development of clinical competencies which, just as the world we live in, constantly change and increase in their complexity.

*Yousef AbouHashem* Faculty of Science and Engineering Macquarie University Sydney, Australia *Manisha Dayal* School of Science and Health Western Sydney University Campbelltown, Australia *Stephane Savanah* Faculty of Human Sciences Macquarie University Sydney, Australia *Goran Štrkalj* Faculty of Science and Engineering Macquarie University Sydney, Australia Email: goran.strkalj@mq.edu.au
